# OpenADR and Agreement Audit Architecture for a Complete Cycle of a Flexibility Solution

**DOI:** 10.3390/s21041204

**Published:** 2021-02-09

**Authors:** Antonio Parejo, Sebastián García, Enrique Personal, Juan Ignacio Guerrero, Antonio García, Carlos Leon

**Affiliations:** Department of Electronic Technology, Escuela Politécnica Superior, Universidad de Sevilla, 41011 Sevilla, Spain; sgarcia15@us.es (S.G.); epersonal@us.es (E.P.); juaguealo@us.es (J.I.G.); antgar@us.es (A.G.); cleon@us.es (C.L.)

**Keywords:** demand side management, flexibility, demand response, advanced metering infrastructure, smart grid

## Abstract

Nowadays, the presence of renewable generation systems and mobile loads (i.e., electric vehicle) spread throughout the distribution network is increasing. The problem is that this type of system introduces an added difficulty since they present a strong dependence on the meteorology and the mobility needs of the users. This problem forces the distribution system operators to seek tools that make it possible to balance the relationship between consumption and generation. In this sense, automated demand response systems are an appropriate solution that allow the operator to request specific reductions in customers’ consumption, offering a discount to the customer and avoiding network congestion. This paper analyzes the implementation and architecture of a demand response solution based on OpenADR standard and its possible integration with a building management system through a use case. As will be analyzed, a key part of the architecture is the measurement system based on smart meters acting as sensors. This is the base of the auditing system which makes it possible to verify compliance with the consumption reduction agreements. Additionally, this study is completed with a parallel auditing system which makes it possible to verify compliance with the consumption reduction agreements. All of the proposed demand response cycle is implemented as a proof of concept in a classroom in the *Escuela Politécnica Superior* at the University of Seville, which makes it possible to identify the advantages of this architecture in the ambit of connection between distribution network and buildings.

## 1. Introduction

The latest advances in the power system are mainly marked by the progressive inclusion of renewable generation, which has significantly increased in the daily energy mix of many countries [1]. Renewable generation is also finding places inside customer facilities, allowing them to produce energy. These customers are known as prosumers (that are producers or consumers according to the moment), and these clients are adopting a more active role within the power system. Moreover, the use of electric vehicles is recently getting more attention, as their autonomy has improved a lot from the first protypes that were seen in the previous decade. 

The problem with these advances is that this type of system introduces an added difficulty in the power system management since they have a strong dependence on the meteorology [2,3] and the mobility needs of the users [4,5]. This uncertainty and variability, together with its increasing deployment of this kind of system, is creating problems due to the overage of the distribution line power capacity. Traditionally, the solutions to this problem consists of a reinforcement of the infrastructure (known as wire solution). However, the required investment would be too large to be assumed by the companies.

In parallel, this problem forces the system operators to seek tools that make it possible to balance the relationship between generation and consumption, i.e., maintaining the unit commitment (the generation must be equal to the consumption). In this sense, the Distribution System Operators (DSOs) are responsible for maintaining the network stability, orchestrating these assets locally as Distributed Energy Resources (DER). This task is critical in sectors of the distribution network that are weakly connected, where congestion management tasks are delicate. 

In this context, Demand Side Management (DSM) and Demand Response (DR) techniques can be a solution to the problem [6,7], allowing customers to provide services of power adjustment when needed. Thus, they can help solve congestion problems as a non-wire solution, reducing their peak consumption, and avoiding oversizing in the distribution grid. Furthermore, they bring the possibility of investment deferral, as they can reduce the overcharge in the most congested power sectors.

In this sense, one of the major benefits is that customer-side resources can transact with the electric grid [8]. In any case, the implication of the prosumer for their participation in DSM and DR programs requires that their facilities become sensorized and connected. Fortunately, after some years, the sensorization of buildings and homes has become relatively easy. Lots of technology and protocols like Wi-Fi smart devices, Z-Wave, ZigBee, Modbus, and advances in computation [9] have facilitated the automation and monitoring at a wide range of levels [10]. These technologies stimulate the extended use of distributed generation, like home solar panels and batteries, which increase the control complexity [11,12]. The users can improve not only the greening of their actions, but also the other high level of the power system, that is, the facilities beyond their homes: the distribution network.

In the European Union (EU), which entity would make this “integration” with prosumers is still being discussed, because it could be done by the Transmission System Operator or TSO (a total centralization), by the DSO with the help of aggregators (in order to respect the mandatory unbundling of the power system), or it could be done in a coordinated way [13,14]. The discussion of these paradigms is not the objective of this paper, but the second one (DSO integration). It will be used for the description of the use case, due to its “lower level” approach, more focused on the possibilities of improving the network at the distribution level.

Following this second class of integration, a prosumer can provide flexibility services to the DSO, helping them make better use of the resources, integrating (in some manner) their resources at DSO network level.

This philosophy is being more and more extended, as can be observed in the new changes in the EU power system regulations [15], where the use of distributed resources is being included under the concept “flexibility”. This concept means the ability to respond in a flexible manner to changes and needs of the power system, using a set of available resources (generation or power reduction). In practice, this is implemented with what is called “flexibility programs”, i.e., agreements between the operator and the prosumers (or even third parties as the aggregators, when it is desirable or required by the regulations [15]) where those prosumers receive incentives (which are processed separately from their contracted electricity tariff) when they successfully respond to the received request (reducing peaks, generation or consumption during a certain period). The mentioned regulation establishes that all customer groups (industrial, commercial, and households) should have access to the electricity markets to trade their flexibility and self-generated electricity, making it possible for aggregators to play a role as intermediaries between these customers and the market. How these flexibility programs and the incentives are handled is still under discussion. As an example, Ref. [16] proposes that the registry and payment of such services could be done using a local market place at the DSO level using technologies such as Blockchain to manage the incentive payments.

In any case, it is necessary to be prepared for these emergent paradigms, where the users and the rest of the power system need to be perfectly balanced and linked, this being an advantage for both parts not only in Europe, but also in the rest of the world [17].

Likewise, there must be a liquidation and review stage later where the compliances with the agreements are verified. Fortunately, within the development that Smart Grids have experienced in recent years, it is worth highlighting the use of Advanced Metering Infrastructure (AMI) [18,19] and in particular the deployment by the DSOs of customers’ smart meters (SM), which following the European directives are fully deployed in most of the European countries [20]. The AMI is composed by two main functions, which are the Automatic Metering Reading (AMR) and Automatic Metering Management (AMM). Both functions are usually integrated in the Smart Metering architecture, which is used for the monitoring of customer consumption and other elements of the power system. In Europe, the deployment of smart metering for gas and electricity started with the publication of the European Directive 2009/72/EC, by pointing out the advantages of such systems, and recommending their deployment [21].

Finally, communication and control of the “internal” elements of a building, as was mentioned before, is solved. There are many technologies and possibilities to integrate sensors, meters, and central control for facilities (even in the case of industrial wide areas, university campuses, etc.). Notwithstanding, communication between the DSO and the user is currently under development and research, mainly since this user-DSO integration is not still fully extended. This final connection between buildings and the distribution network is sometimes referred as “the last mile” of connection, as can be seen in Ref. [22].

Specifically, the work described in this paper is focused on this “last mile” environment. The objective is to define a complete system architecture for prosumers, providing a fluid communication channel with the aggregator/operator and capability to participate in flexibility services, including the audit mechanism. This proposal has been deployed on a testbed, which is called “DERBis lab” here, which is made up of a DR architecture, a Building Management System (BMS) to manage and operate the different loads, and an AMI solution to validate the compliance with the agreements between the different parties. The communication channel with the DSO/aggregator uses the OpenADR standard. Additionally, the smart metering infrastructure is based on the PRIME standard.

In this paper, the whole process of DR planning is analyzed from the point of view of the customer, including the use of their own sensing and control systems to check the flexibility services availability. The architecture and process steps are described, analyzed, and finally tested using real data and information from the BMS and the smart metering devices. It is remarkable that the proposed architecture has been designed considering the same type of smart meters that are already deployed in most countries, which perform hourly consumption measurements. This restriction has been applied in order to avoid the need of deploying new metering systems for the present application, which would lead to higher costs.

Before describing the proposed solution, a brief explanation about the state of the art in the field of flexibility and DSM implementation and AMI solutions is carried out in Section 2. Section 3 contains the description of architecture and its parts. Then, Section 4 details the implementation of the proposed architecture for the DERBis lab. Section 5 shows three study cases of flexibility services. Finally, the conclusions are shown in Section 6.

## 2. State of the Art

The previous section outlines how the inclusion of flexibility programs could potentially bring multiple benefits for the management of the power system. The next step is to discuss which of the possible architectures is the most convenient one for the inclusion of prosumers, and how their possible actions should be categorized for their scheduling and dispatching.

The tools for power management in the level of unit commitment are classified in different ways depending on the zone. In Ref. [23], J. Ortega, D. Watts, and H. Ren review how the operating reserves are categorized in USA, Chilean, and German markets. They conclude that five categories are used in the case of America, while three of them are established in Europe.

From the list of European categories, the one that can be considered closer to the flexibility from the customer side is the Tertiary Reserve. This reserve, which operates a maximum number of times in 15 min, is manually activated. Its use is a response to a disbalance in the control area and/or bigger congestion problems [23]. This description corresponds to their use in the context of system operations, but the same idea can be applied in congestions at the DSO level, even considering longer times (hours) for dispatched DSM events. In the context of American power markets, these services can fit into the category of Supplemental Reserves.

There are, in fact, some examples of North American distribution companies that have already included DSM collaboration for their customers. This is the case of the companies San Diego Gas & Electric (SD-G&E) [24] or Pacific Gas & Electric (PG&E) [25], whose DR programs are focused on what is called a “Capacity Bidding Program”, where a customer commits, under contract, to reduce their power a certain quantity when required. The maximum number of requests is also established in the agreement. Other approaches propose the organization of flexibility and ancillary services under a specific market where TSOs and DSOs could buy those services that they need from prosumers and aggregators [26].

According to [6], DSM techniques can be categorized according to their objective. The categories are direct load reduction, load scheduling, pricing schemes, based on optimization types, and home energy management.

Fortunately, in recent years some technology and protocols have emerged to solve these connections. Not only for DR, of course, but for managing the global Smart Grid environment, where DR is usually included. This has been a natural response to the rising environmental concern around the world, particularly in the U.S., Europe, and Asia [27].

A review of DR is presented in Ref. [28]. Specifically, this last paper studies the advantages of Automatic Demand Response (ADR) for DSM. ADR is the implementation of DR using Information and Communication Technologies (ICTs) without needing manual interventions. Samad et al. states that ADR is critical technology in DR. Additionally, in order to implement ADR, standard communication protocols such as OpenADR [29,30] are essential to allow interoperability as the authors state.

OpenADR is a protocol to allow remote ADR procedures. Despite this protocol being very “general”, it offers many possibilities for running DSM and DR programs [31] in a variety of possible architectures. Some examples of these options are exposed in Refs. [32,33], putting special emphasis on the different architectures and connections that could be used. Another good point of this protocol is that it includes functions for dynamic pricing, which are used for example in Ref. [34] as part of more complex Smart Grid managing system.

This is not the only service that can be included in the DR category. Another example of an ancillary service is peak saving [35]. The use of DR is not limited to the domestic/residential level, and examples at the industrial level can be found. An example is [36], where a service of DR is applied to refrigerated warehouses.

The participation of a customer in DR implies two steps: the event dispatching, and the audit of the actuation during such an event. This means that the distribution company must check if the customer accomplished the requirements of consumption during the established time.

Thanks to the network modernization drive by international directives (i.e., European directive 2009/72/EC [21] in which the energy smart meter deployment program was established for the first time), this audit can be done using the same systems that are usually used to establish the power bill, which are the customer’ smart meters. As [28] states, although ADR can be conducted without them, smart meters can enhance the implementation of a DR program, as they enable more elaborate means of compensating for DR participation by establishing baselines and comparing demand-side performance against those baselines. An example of use of OpenADR and smart metering can be seen in Ref. [37].

**Figure 1:**
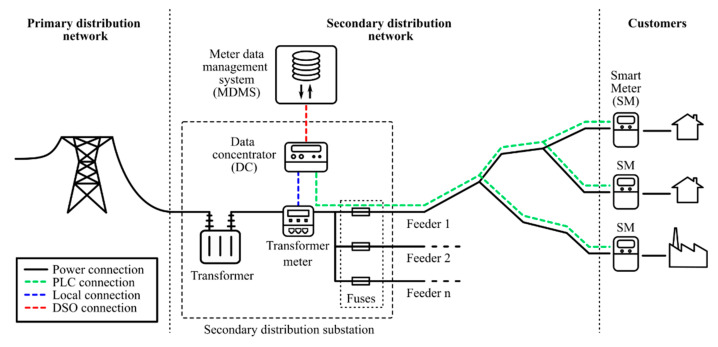
PLC (Power Line Communication)-based smart metering infrastructure [38].

There are many types of solutions for the deployment of smart metering infrastructures, one of the most important characteristics being the method of communication. While non-wired solutions have many advantages, the Power Line Communication (PLC) solutions are most extended for electricity metering, as they use the power lines as communication channels. A general structure of a PLC smart metering infrastructure in the power system can be seen in Figure 1.

As mentioned in the previous section, the deployment of smart metering is relatively advanced in Europe thanks to the impulse provided by legislative powers. This is very convenient for the future of ADR, as it can serve as auditory infrastructure. In fact, the smart metering deployment is very advanced not only for the electric sector, but also for water and gas utilities [39].

However, currently, the smart meter architectures are traditionally operating with aggregated measurements of one hour. Therefore, if DSOs want to use this system for auditing compliance with flexibility agreements, the offers and actions must match these time intervals. This limitation would be reduced if this granularity became a quarter of an hour, a possible period for this type of network, and more in line with flexibility services. 

The conditions of the audit should be based on the expected or estimated consumption of the customer during the period of the event, which will be compared to the actual one to check if the power was effectively reduced or not. In the case of buildings, this estimation can be done using multiple techniques, based on statistical, or even artificial, intelligent methods [40,41].

The review of the state of the art regarding the functionalities and applications of DSM, DR, and Smart Metering has shown that their use can bring multiple benefits to the power system. Nevertheless, their deployment brings about various technical complications, such as the integration and coordination of the different systems and actors. The next section proposes an architecture pointing out the requirements of each of its parts for DSM/DR implementation.

## 3. Proposed Architecture

**Figure 2:**
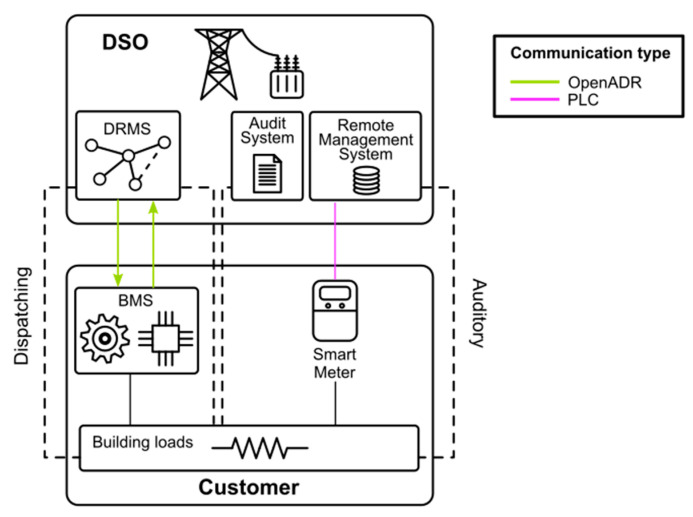
Structure and communication of DSO and customer systems for DSM/DR and auditing.

In this section, the proposed architecture is described. This architecture includes the complete cycle of DSM, including dispatching and auditing. The general structure is the one shown in Figure 2.

As can be seen in the Figure 2, the DSM cycle is split into two parts: dispatching and auditing. The dispatching is done through the OpenADR protocol while the auditing is done through the AMI based on PRIME standard. The system in charge of managing the customer flexibility services is the BMS.

The proposed architecture aims to take advantage of the already existing metering system for the audit process, not requiring any remarkable economic effort. The automation systems would require an update to fulfill the requirements, but there is currently a rising tendency to install BMSs in big and medium size customers and domestic automatization systems, so it is expected that these systems will become relatively common and its integration with the proposed approach will be easy. Finally, it is true that it is the DSMs architecture which requires bigger changes for its integration in the DSO control systems. Notwithstanding, it is a clear interest by the DSO. Proof of that is that various DSOs are already exploring the use of this kind of techniques, citing as examples the initiatives of San Diego Gas & Electric Company [24] and Pacific Gas & Electric Company [25].

Therefore, three main parts can be distinguished from the customer side, the DSO-user communications (DRMS-BMS) through the OpenADR protocol, the BMS in the customer side, and the smart metering system to audit the DSM operations. This section attempts to analyze, in detail, the required characteristics of the three parts. Moreover, the Section 3.4 explains how the whole process of DSM dispatching and auditing works.

### 3.1. OpenADR Standard

As previously stated, a crucial aspect of flexibility (and, more specifically, DSM) and, therefore, congestion management is the communication between service providers (or customers) and the DSO. In this sense, the development of OpenADR standard is becoming an important tool to communicate grid flexibility actions.

OpenADR came up in 2002 at the Demand Response Research Center, which is part of the Lawrence Berkeley National Laboratory (LBNL) in California [30]. In 2009, OpenADR specification (v1.0) specification was donated to different standardization organizations including the Organization for the Advancement of Structured Information Standards (OASIS) turning into an open-based specification. In 2010, the OpenADR Alliance was created by industry stakeholders to support the development, testing, deployment, and standardization of this protocol. The OpenADR 2.0 standard, which was released in its first version (2.0a) in 2011, was created using a profile from the Energy Interoperation version 1.0 standard from OASIS, which is an information model to enable collaborative and transactive use of energy. Currently, it is in version 2.0b since 2015. The main difference of this version with the previous one (2.0a) is that the current version includes four services inherited from the Energy Interoperation (see below for the description of the services) while the previous one only includes one service (the EiEvent) and it is limited to just one signal.

OpenADR is an open specification designed to facilitate and automate DR communication [42]. It is a communication data model, but it also defines transport and security mechanisms. The protocol is based on eXtensible Mark-up Language (XML). As transport mechanisms, OpenADR uses Extensible Messaging and Presence Protocol (XMPP) or Hypertext Transfer Protocol (HTTP) using REpresentational State Transfer (REST).

The protocol defines two kinds of actors or nodes inherited from the OASIS Energy Interoperation, the Virtual Top Node (VTN), and the Virtual End Node (VEN). Communication is hierarchical, always between a VTN and one or more VENs. Thus, no communication between the same kind of nodes could take place on an OpenADR schema. Nevertheless, a node can be at the same time a VEN and a VTN of another hierarchically lower OpenADR stage. In an OpenADR schema, the VTN is responsible for communicating the DSM conditions and other relevant data to other entities (VENs). In this sense, the VEN is responsible for controlling and managing the electrical resources at the final consumer level or aggregating different resources and/or clients. Thus, the VTN will normally be at the DSO side and the VEN will be at the customer side.

The VENs can be either physical devices or a cloud-based platform. Physical devices can have direct control of the customer assets based on the flexibility commands coming from the DSO. Cloud-based platforms can be used by Flexibility Service Providers (FSP) or Aggregators to gather different assets from different locations and owners in one single endpoint making the interaction easier for the DSO. As previously mentioned, the current regulation does not allow the DSO to own DER assets or to communicate flexibility signals to final customers in Europe. Nevertheless, flexibility signals to a customer using a third party like an aggregator are allowed.

**Figure 3:**
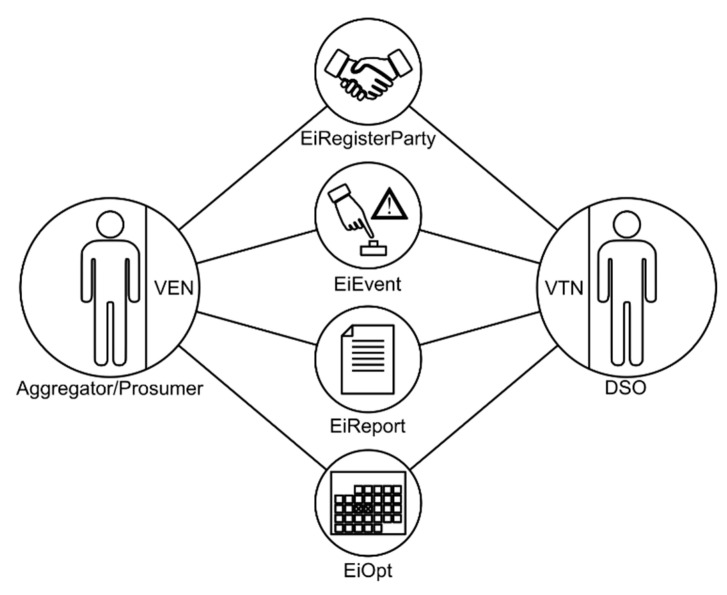
OpenADR communication services.

The current version of OpenADR defines four main communication services (see Figure 3) inherited from OASIS Energy Interoperation:

- EiRegisterParty: Used to identify and enable communications between a VTN and a VEN.
- EiEvent: Used by the VTN to request DR operations to one or more VENs. In the OpenADR terminology, the process of requesting a DSM is called event. Events can be accepted or rejected by the VEN.
- EiReport: Used to share data. Both the VTN and the VEN are able to report information to each other, e.g., the VEN could report metering information to the VTN.
- EiOpt: Used to communicate availability or unavailability, e.g., a VEN can communicate the period of time in which it will not accept any events coming from the VTN.

In HTTP, VENs can communicate either in pull mode or push mode. In push mode the VEN acts as a client and the VTN acts as a server, the VEN periodically polls the VTN to see if it wants to communicate anything. In push mode, both the VTN and the VEN can act either as server or as client, thus, communication can be asynchronous, and no polling is needed. In a XMPP schema, due to its nature, communication is asynchronous, and no polling is needed.

Additionally, OpenADR defines a fifth service: the oadrPoll, which is only used by the VEN in the HTTP pull mode to periodically poll the VTN.

#### VTN Development

**Figure 4:**
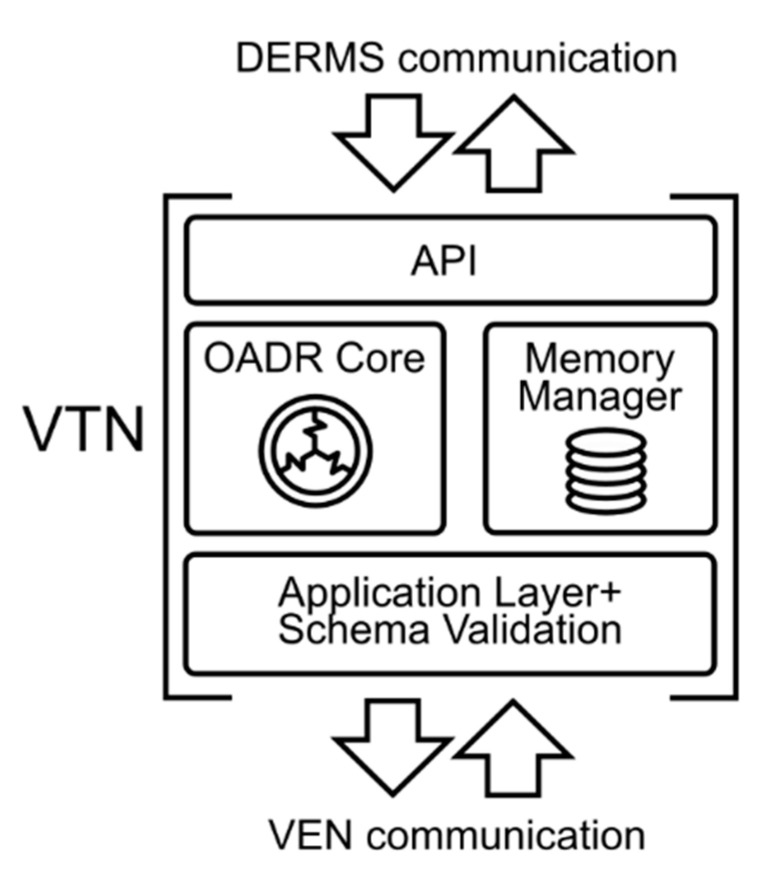
Architecture of the developed VTN with the different layers.

There exist multiple implementations of VTN software [43], but they do not have an external interface to enable interoperability with other Smart Grid systems like a Demand Response Management System (DRMS) or a Distributed Energy Resources Management System (DERMS). Therefore, a VTN has been developed by the authors to fulfil these requirements. The architecture is shown in Figure 4. The VTN architecture is divided in four main modules. From bottom to top:

- Application Layer and Schema Validation: It handles the OpenADR HTTP services. The VTN is pull and push mode compatible. In addition, the compliance of the messages with the OpenADR protocol is also checked.
- OpenADR Core: It implements the OpenADR 2.0b services.
- Memory Manager: The memory manager is split into a Cache Memory manager which stores frequently requested data in RAM and a Persistence Memory Manager, which manages the connection with a relational database. This split allows the reduction of the database access, increasing the response times of the system.
- API: It provides an interface to other systems to use the VTN services. It is based on REST and JavaScript Object Notation (JSON).

The data models used in the VTN infrastructure, besides OpenADR, also obey the Common Information Model (CIM) from the International Electrotechnical Commission (IEC). OpenADR is harmonized with this standard, which means that it is interoperable with Distribution Management (IEC 61968) and Energy Management (IEC 61970). This, together with the API, makes the VTN interoperable with other Smart Grid systems.

Thanks to the API, the VTN acts as a gateway for other Smart Grid systems. In this sense, the VTN does not take any real actions by itself. These actions are done by means of the API interface. Additionally, the layer-based architecture makes the system easily upgradable to other OpenADR versions, even the EI.

### 3.2. Building Management System

A BMS is a system that centralizes the information and control of a building or area. Their possibilities go from the mere monitorization of the consumption or comfort conditions to the advanced control algorithms and automation (e.g., [44] that propose a local Energy Consumption Scheduling for HVAC control that which compensates for possible errors between weather forecast and real local conditions). In this sense, most of the BMS control approaches are focusing on getting improvements in energy efficiency [45], obtaining the optimal use of renewable resources [46], or are based on static pricing schemes [47].

Beyond these local or static approaches, in the context of their inclusion as part of a DSM cycle, they should also accomplish certain tasks to permit the participation of the customer resources in flexibility services.

Firstly, it must be aware of “when” and “how much” the resources can provide power reduction (or extra generation) if required.

Secondly, it should include any type of consumption prediction, at least in a horizon of one day, to calculate which is the effective “margin of flexibility” that the building can offer.

Finally, it must count with the communication systems that are used for the flexibility market and the DSO. This point is related to what was previously exposed regarding OpenADR. Thus, the BMS needs a communication endpoint with the DSO as means of an OpenADR VEN.

### 3.3. Smart Metering and Audit Process

A smart metering system is made up of smart meters (usually one for each client) and a data concentrator, which receives the measurements and sends the information to the DSO central systems. The communication can be performed in multiple ways, but one of the most popular ones is the PLC [48]. This communication is done through the power lines, so it does not require extra elements for the deployment of smart metering.

About the protocol for the PLC, some of the most extended ones in Europe are:

- PRIME: Powerline Intelligent Metering Evolution (PRIME). Defined by the “Prime Alliance”.
- Meters&More: Defined by the “Meters and More Association”.
- G3PLC: Supported by the G3PLC Alliance.

PRIME will be the chosen protocol for the present proposal, it being one of the most extensive in Spain. Notwithstanding, any of them could be valid from the point of view of required functionality. Thus, as was commented before, the consumption measurement period for billing is typically hourly in Europe. This measurement interval could initially be considered adequate for this application, although it would be advisable to increase the resolution to a quarter of an hour, in order to better adapt to the requirements of flexibility services.

### 3.4. Complete Flexibility Cycle Process

**Figure 5:**
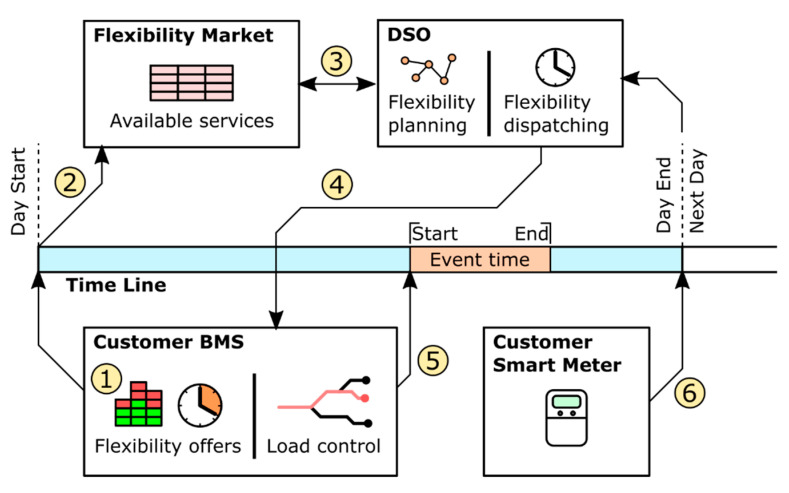
Complete flexibility cycle. (1) Flexibility offers calculation [BMS]; (2) Send offers to market [BMS-Market]; (3) Services selection [DSO-Market]; (4) Service request [DSO-BMS]; (5) Load control for requested service [BMS-Loads]; (6) Auditory of the requested service [DSO].

Figure 5 presents a resume of the whole process of flexibility estimation, participation in the market, event receiving and execution, and the audit.

At the start of the day (or before, depending on the flexibility program), the BMS performs an estimation of their own consumption (and generation, if any), and also considers which devices are curtailable (Stage 1 of the Figure 5). The flexibility offer is derived from these values, which is the description of the possible load reduction for each of the hours of the day (Stage 2). This offer is sent to the flexibility market, where all the offers of all the customers under the market are listed (Stage 3). The DSO selects those offers that are needed for the flexibility planning, and their selection is communicated to the affected customers using an OpenADR event (Stage 4). These events can be sent along the day but should be done some time before the moment when the requested event will start (e.g., at least one hour before). This restriction is usually described in the flexibility program. When the time of the event starts, the BMS will manage the loads to keep the requested power constraints according to the event characteristics, until the event finishes (Stage 5). At the end of the current day (or at the end of the settlement cycle), the DSO will have retrieved the data from the smart meters and make the audit for the billing adjustment, depending on whether the customer had correctly achieved the request of the event (Stage 6).

In the next Section, the implementation of these systems in a real laboratory is detailed.

## 4. Laboratory Systems

To demonstrate the correct operation of the system and the benefits of OpenADR in flexibility operations and congestion management a use case has been conducted. This use case has been conducted using the accommodations at the “DERBis” located at the *Escuela Politécnica Superior* of the University of Seville. The DERBis is a demonstrator of Smart Grid, DERs and Building Management technologies.

### 4.1. DERBis Description

The classroom has 23 seats (22 for students and 1 for the professor), each one with a PC. This space is located at the *Escuela Politécnica Superior* and it is mainly used for teaching purposes for the MSc degree in Intelligent Systems, Energy, and Transport.

Nevertheless, this classroom has become a living lab for different Smart Grid and Building Management technologies. This facility makes it possible to train students in the different possibilities of these technologies and how to manage them. Additionally, this space is used by researchers to test different scenarios and use cases related to flexibility.

**Figure 6:**
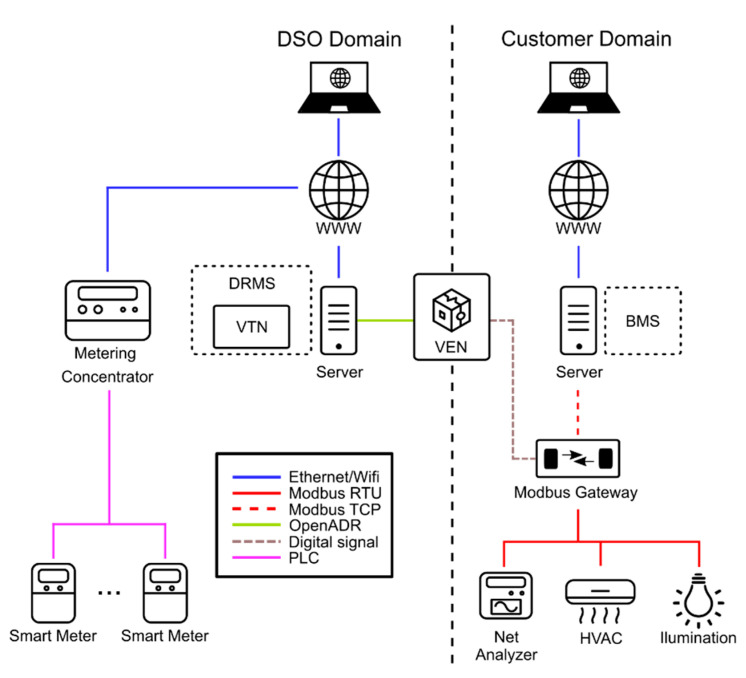
DERBis systems overview. DRMS: Demand Response Management System; VTN: Virtual Top Node; VEN: Virtual End Node; BMS: Building Management System.

The complete overview of the different devices and systems deployed in the lab is shown in Figure 6.

**Figure 7:**
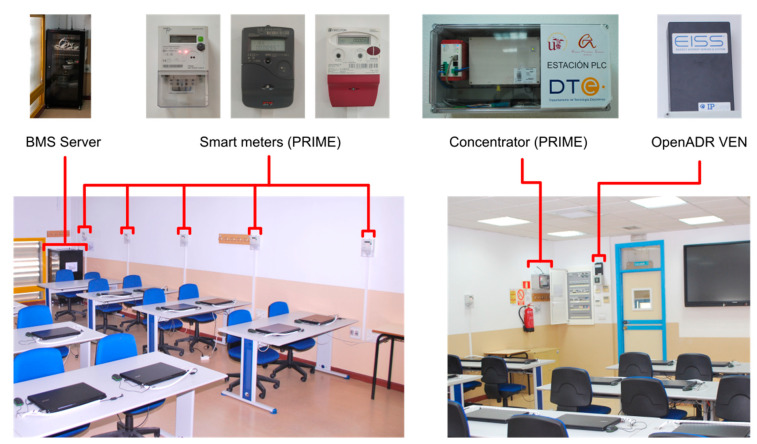
DERBis control and measurement devices.

On the one hand, the current Smart Grid deployments in the lab are an AMI based on smart meters and a physical OpenADR VEN. On the other hand, the Building Management deployments are based on different controllers with Modbus communications, it being possible to control different systems, such as: The Heat, Ventilation and Air Conditioning (HVAC) modules, the light circuits, and the user’s loads (wall plugs). Additionally, a centralized system manages all the resources of the lab. This system acts as a BMS that controls the room assets based on different rules and constraints. Figure 7 shows the devices inside the room.

Thanks to the controllable loads, and based on the energy demand prediction, the lab can provide flexibility services despite not counting with generation resources, simply reducing the load when it is required.

#### 4.1.1. Smart Grid Technologies on the DERBis Living Lab

Additionally, a physically OpenADR VEN has been installed: an EISSBox 3.0 [49] (see Figure 7). This VEN is fully OpenADR 2.0b compatible and it is currently connected to the VTN developed by the authors. Through this VEN, OpenADR commands can be sent to the lab simulating DSO actions. Also, the consumption of the lab can be obtained from the VEN by means of the report service of the OpenADR protocol. 

This VEN has four relay outputs that can be configured to respond to OpenADR events. Also, the VEN has four pulse inputs for energy metering purposes. Two of the relays of the VEN are connected to an input Modbus device in order to read their state. Two of the pulse inputs of the VEN are connected to the pulse outputs of the Modbus energy meter.

The smart meters of the DERBis use PLC communications based on PRIME protocol. All smart meters communicate with a PLC concentrator (CIRCUTOR SGE-PL1000) which receives the measurements and can send different commands to the meters. These commands are related to the management and configuration of each smart meter, which can be done separately. A more detailed description of the AMI devices can be found in [38], where their characteristics, structure, functions, and possibilities for teaching purposes are pointed out.

**Figure 8:**
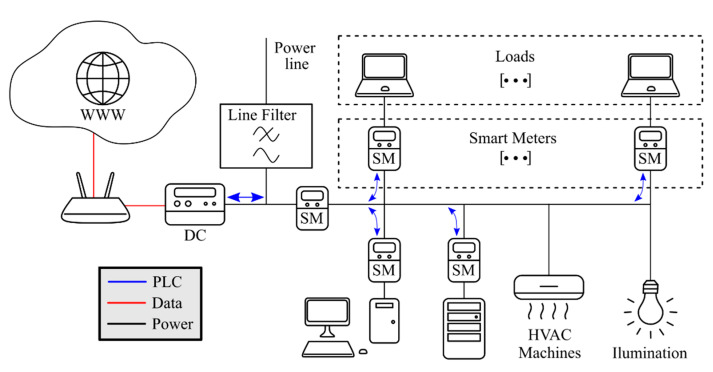
Smart metering system.

The Smart Meters are distributed through different loads and locations of the lab (student benches, HVAC devices, etc.). In addition, one Smart Meter is installed at the head of the lab, measuring the overall room consumption. The schema of the smart metering system can be seen in the Figure 8.

#### 4.1.2. Building Management Technologies on the Living Lab

From the Building Management point of view, the lab has different Modbus RTU devices for control and monitoring HVAC, lights and loads. To integrate all these devices with the BMS, a Modbus TCP/RTU gateway has been installed.

Specifically, the lab has three HVAC controllable machines. These machines have a proprietary remote configuration protocol. In this sense, one of these machines is fully controlled by means of a gateway between Modbus RTU and its proprietary protocol. The other two only have an on/off control. The lights and users’ loads also can be monitored and have an on/off control. Additionally, different electrical parameters (voltages, currents, active and reactive power, Total Harmonic Distortion (THD), etc.) of the lab can also be obtained by means of an energy analyzer. Ambient conditions are also monitored by a temperature and humidity sensor.

**Figure 9:**
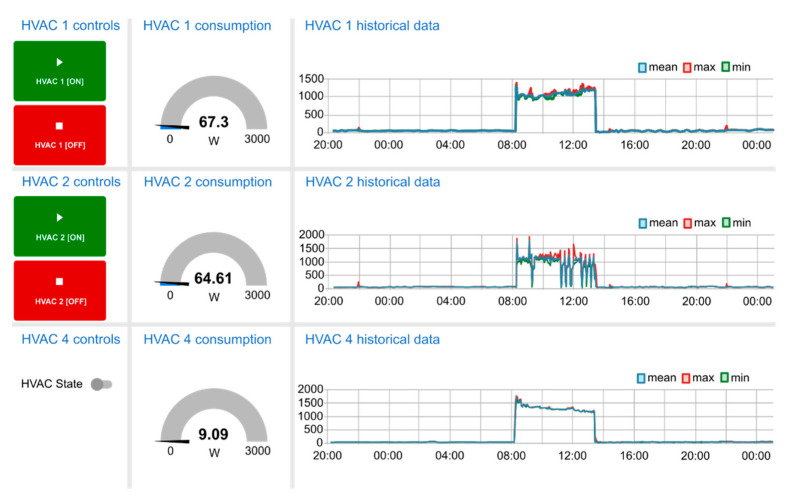
BMS HVAC control interface.

All these systems are controlled by the BMS which integrates all the devices and systems. The BMS offers monitoring and historical services of the Lab and also implements different control schemas to improve and adjust the energy consumption. Additionally, it is the responsibility to set the appropriate adjustments when requested by the DSO by means of the VEN. A capture of the BMS interface can be seen in the Figure 9.

As can be seen, this location offers a great integration of technologies from both Smart Grid and Building Management. In this sense, this space provides a great opportunity to test the interaction between different systems and technologies as well as to test different flexibility operations in the scope of the congestion management in the Smart Grid.

## 5. Use Cases

Thanks to the great opportunity that this living lab offers in terms of technologies and integration, several use cases have been carried out. These use cases expect to test the living lab capabilities, the OpenADR protocol schema in a real scenario, the auditory process through the AMI as well as the developed systems for grid flexibility operations, especially in congestion management.

The proposed schema will involve a DSO operation request for a customer (or an FSP/Aggregator) to mitigate a congestion in the grid. In this sense, the developed DRMS/VTN will act as the DSO system, and the actions that are supposed to be done by the rest of the DSO systems (Distribution Management Systems (DMS), Energy Management Systems (EMS), etc.) in the DRMS/VTN will be simulated. Although, thanks to the interoperability with different standards that the VTN offers, the connection with the DSO could be easily done.

The DERBis living lab will act as the assets on the customer side. Thus, the OpenADR will be the communication link between the DSO and the customer. In this sense, the VEN located at the lab will be the endpoint on the customer side. Then, the BMS receives the information from the VEN and performs the required management tasks regarding the events and load management.

Furthermore, the DSO could also get the metering of the lab consumption by means of the AMI deployed with the Smart Meters. Even though OpenADR allows one to send metering through the report service, the DSO also has the metering through its AMI infrastructure with which it could audit the fulfilment of the flexibility command. This is an important point in flexibility for the DSO, since with the smart meter infrastructure has an external way to audit the operation.

It must be remarked that the Smart Meter system is considered here the most effective and adequate system to realize the auditory process. The VEN metering capacity constitutes just an additional auxiliary method which can be used for the measurement of specific parts of the consumption/generation, achieving a more complete understanding of the state of the customer systems, but should never be used for audits.

A general schema of the tasks to be performed by the DERBis BMS is:

1. Start of the day:
  - Estimate power (using occupational model).
  - Include controllable loads (if any).
  - This result in the maximum and minimum values for each of the hours of the day.
  - Calculate baseline (mean of consumptions of previous days for each hour according to the DSO rules).
  - Calculate flexibility margin (baseline minus maximum expected consumption).
  - Send participation offer to the flexibility market.
  - The DSO evaluates if they are interested in sending any event and informs the customer.
  - The BSM receives the event information.
2. Event start (if any):
  - The BMS control the loads (if necessary) to accomplish with the event.
3. End of the day and in advance:
  - The audit of the service requested by the DSO will be done. If the audit power minus the actual power is greater or equal to the event reduction power, the customer has successfully performed the requisites.

Once the process to be followed for flexibility prediction and planning is exposed, some study cases will be exposed. The first one corresponds to a day where there are no curtailable loads, but some flexibility services can be provided thanks to the expected use of the lab during that day. The second one considers some controllable loads to provide the service of power reduction during various hours. Finally, the third one presents a day when there is a very low flexibility margin during one of the hours, so it is considered non-sufficient for participation in the market. Of course, the consideration of a “minimum” power of reduction will depend on the preferred strategy for the BMS, on the conditions of the market, or on the requirements of the corresponding DSO for each day. In the third example a minimum limit of 500 W was considered specifically for that day. It is not the objective of this paper to discuss the details of the market coordination. Therefore, for the present study the events have been launched using the mentioned VTN (which corresponds to the actions of the DSO).

### 5.1. Use Case 1. Absence of Curtailable Loads

**Figure 10:**
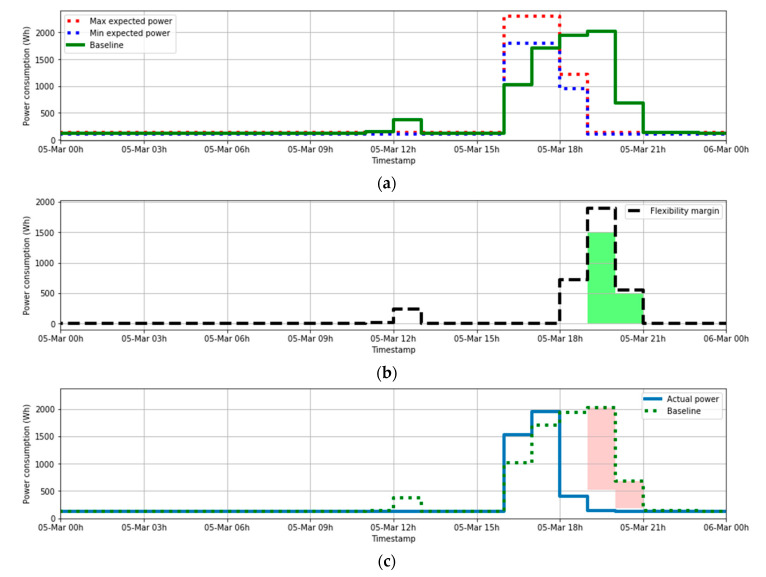

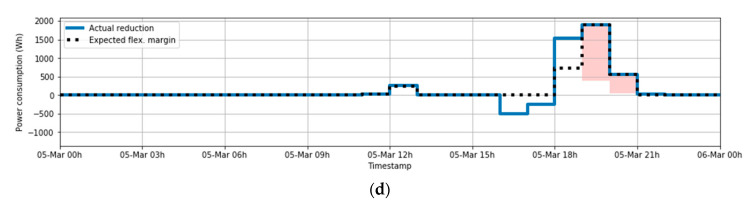
(**a**) Occupancy-based (from BMS) and historical-based (baseline) expected power; (**b**) Expected flexibility Margin (only values >0). Reduction really requested by the DSO (in green); (**c**) Actual power and baseline. Requested power reduction (in red); (**d**) Reduction achieved vs. Expected flexibility margin. Requested power reduction (in red).

The BMS must perform some tasks to manage the flexibility capacity of the laboratory. Firstly, it takes the historical data from smart meter telemetry and applies the rules that are considered by the DSO. In this study case, this baseline will be estimated through the mean power for each hour using the two previous days of the same type (weekend, or non-weekend types). The second task is performing a prediction of the expected power consumption according to their own models (not necessarily based only in previous data, but also including other types of available data). In this case, a simple model based on occupancy has been applied. This model gives a margin of maximum and minimum expected value for each hour and the flexibility margin is calculated as the difference between the maximum expected consumption and the forecasted power. As an example, the values associated for the day 5 March 2020 can be observed in the Figure 10a.

In this sense, under atypical laboratory use, the flexibility margin can be positive, providing potential opportunities to participate in any DR event. Based on this information, the BMS will choose their participation or not offer a reduction in that period.

Thus, in the case of a party agreement, it will be necessary to perform the audit. At the end of the day the smart meter data of the real consumption for each hour will be available for the DSO. Therefore, from there (at any moment in advance) the DSO can make the audit to check whether the lab (that acts as customer) has really accomplished the reduction in the events that they promise, in order to apply the corresponding bill adjustment. 

For the present example, the flexibility margin can be calculated as the Baseline minus the maximum expected power consumption. Considering the flexibility margin, the DSO could pick up from the market the reduction needed. The Figure 10b shows (in green), which was the request from the DSO. It can be noticed that this request is always equal to or less than the flexibility margin (which was the maximum proposed reduction that the BMS estimated).

Figure 10c shows the comparison between the actual power consumption and the audit power. As can be seen, the reduction that was requested by the DSO from 19:00 to 21:00 was successfully achieved.

The Figure 10d shows the comparison of the actual reduction achieved by the customer and the originally expected flexibility margin. The reduction event requirement appears in red. In those hours where the flexibility margin was expected to be positive, a reduction was achieved. Therefore, the prediction of the possible participation of this customer in DR events was correctly done, showing the best hours for their inclusion in these events.

### 5.2. Use Case 2. Controllable Loads Available

In the previous section, the exposed example of load reduction was performed without any controllable load action.

In this second example, the flexibility providing is achieved by using some controllable loads, which are the HVAC system of the laboratory. The process of estimating the flexibility margin is very similar to the one performed in the previous case. The only new elements that are introduced here are the parameters of “controllable load availability”, i.e., the power that can be put off for each of the hours of the day, estimated in a 24 h horizon (at 00:00 of each day). The next data corresponds to the day 26 February 2020.

**Figure 11:**
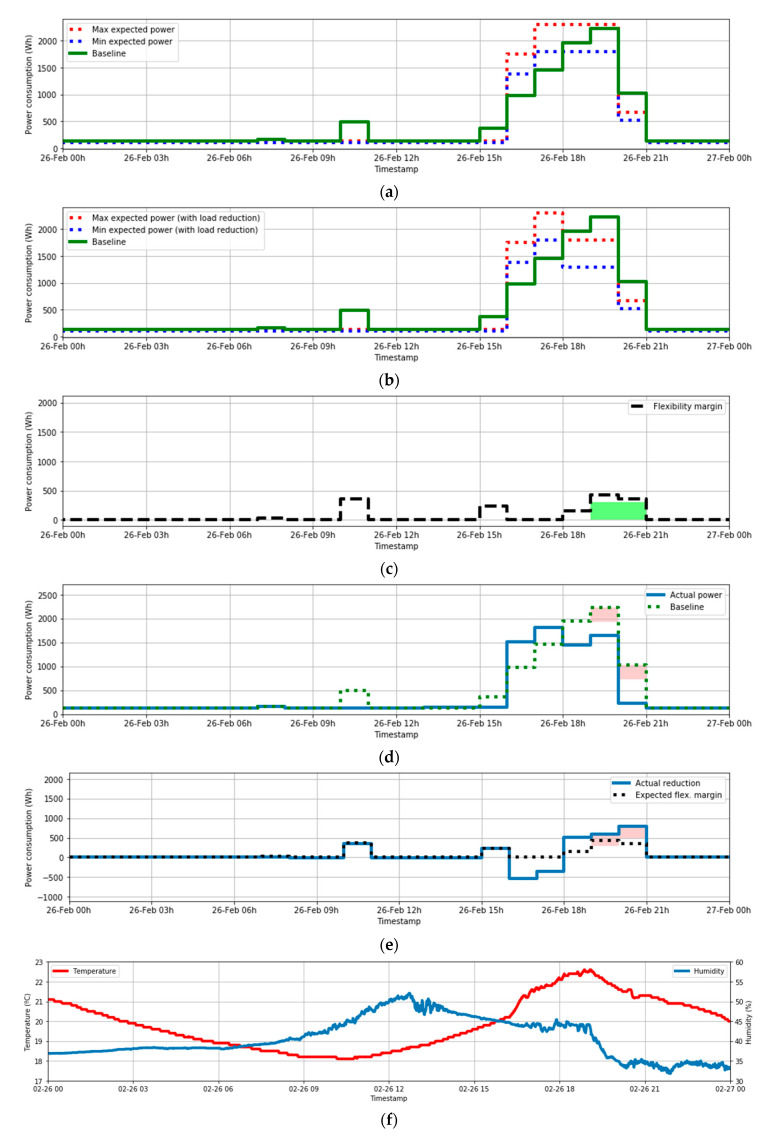
(**a**) Occupancy-based (from BMS) and Historical-based (baseline) expected power; (**b**) Occupancy-based (from BMS) and Historical-based (baseline) expected power—Load reduction considered; (**c**) Expected flexibility Margin (only values > 0). Reduction really requested by the DSO (in green); (**d**) Actual power and baseline. Requested power reduction (in red); (**e**) Reduction achieved vs. Expected Flexibility Margin. Requested power reduction (in red); (**f**) Temperature (red) and humidity (blue).

The expected power without considering the presence of controllable loads can be seen in the Figure 11a.

In this case, according to the BMS estimations, the HVAC system could be deactivated during the period 18:00–20:00 without affecting the comfort conditions of the laboratory. Therefore, this reduction is included in the Figure 11b. The reduction was about 500 W; therefore, this quantity of reduction is applied to update the maximum and minimum expected power.

Using this estimation and the auditory power (which is the baseline power according to the DSO for the auditory process), it is possible to obtain the estimation of flexibility margin as the difference between the auditory power and the maximum expected power. This is done in Figure 11c.

This flexibility margin marks the maximum flexibility offer of the customer for the day. Let’s suppose that the only period where the DSO buys the flexibility services is during the day 26 February from 19:00 to 21:00. The BMS will deactivate the loads during this period (at least). The actual consumption during the day is shown in the Figure 11d.

The comparison between the actual power and the audit power (baseline for auditory process) shows that the reduction was successfully achieved during the period of event can be seen in Figure 11e. The reduction requested appears in red.

Finally, the data of temperature and humidity are shown in Figure 11f. It shows how these two variables start to change their tendency due to the disconnection of the HVAC system, but they maintain adequate values during the reduction event in time of use of the laboratory, keeping good comfort conditions for the users.

### 5.3. Use Case 3. No Significant Flexibility Margin

The third situation that can occur is that the BMS does not find any flexibility margin during the day (or simply that this margin is too low). This means that the customer should not participate in the flexibility market, as they are not expected to be able to offer any load or generation adjustment.

Therefore, the action of the BMS will finish in the calculation of flexibility margin, not offering any reduction or attending any event during the day.

**Figure 12:**
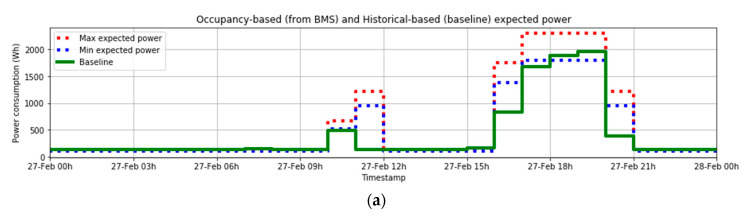

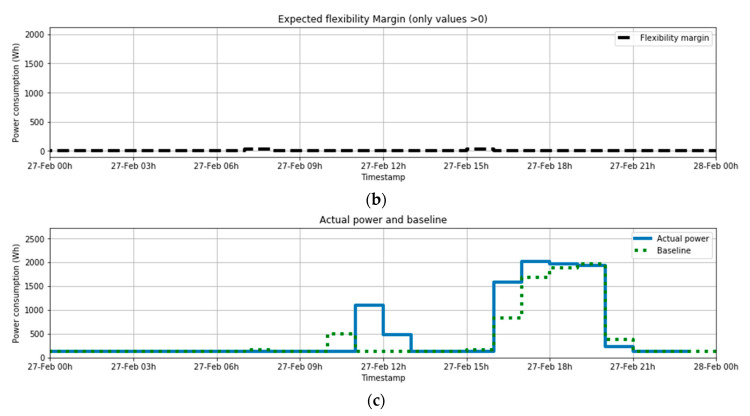
(**a**) Occupancy-based (from BMS) and Historical-based (baseline) expected power; (**b**) Expected flexibility Margin (only values >0); (**c**) Actual power and baseline.

An example corresponding to the day 27 February 2020 is shown in Figure 12a. On this day, there is no hour when the flexibility margin is significant, but only some residual values (see Figure 12a,b). Therefore, participation in the flexibility market is not possible to be considered, so the BMS will not send any offer.

Figure 12c shows the actual power throughout the day and the baseline. Of course, as the customer has not participated in the flexibility market with any offer, there will be no audit process referred to this day.

Throughout the three cases analyzed, how the BMS performs the previsions of flexibility margin in order offer a possible reduction to the DSO can be observed. In some of the days there will not be any possibility of providing any service, while in others it will be possible thanks to the use of controllable loads, or thanks to the analysis of the expected occupation. 

## 6. Conclusions

The inclusion of DSM over the power system undoubtedly constitutes a powerful tool to improve the management capacity and flexibility of distribution networks. However, the use of these kinds of services implies an agreement between the two parts, i.e., the customers and the DSO, which could be performed under multiple existing terms, principles and methods. 

In this paper, a possible architecture for DSM dispatching and audit is proposed and tested. In this architecture, all the controllable devices are connected to a BMS. As it was exposed, the BMS established communication to the DSO systems using the standard OpenADR, providing a channel to inform about availability, scheduling, prices or any needed data.

The audit function depends on the DSO smart meter infrastructure that provides remote information about the hourly consumption, this data being used to check the behavior of the loads in question during the period of the dispatched DSM events. The accomplishing or not of such events will therefore be evaluated using information from the smart meters to perform the audit process. Likewise, this hourly information is enough for this purpose, although its extension to a quarter hour is considered interesting for the future to increase network flexibility.

As Proof of Concept the architecture has been tested on a real testbed located in a laboratory of the *Escuela Politécnica Superior* of the University of Seville. In this sense, as can be seen in its results, the proposed architecture clearly shows the possibilities of use of controllable loads under the established DSM contract conditions. The principal difficulty from the customer side would be the requirement of the OpenADR VEN device, which would not be such a big problem in the case of buildings that count with a BMS. Thus, thanks to taking advantage of the available power meter infrastructure that is used for the electricity billing, the audit process can be done by the DSO in a secure and appropriate manner, showing the adequacy of the AMI for this task, as this system makes easier the integration of flexibility services.

It can also be concluded that the described laboratory constitutes a complete platform where some of the most important smart grid related technologies can be evaluated, integrated and taught.

## Data Availability

The data presented in this study are available on request from the corresponding author. The data are not publicly available due to project privacy issues.

## References

[1] Gerard H., Puente E.I.R., Six D. (2018). Coordination between transmission and distribution system operators in the electricity sector: A conceptual framework. Util. Policy.

[2] Mohammed N.A., Al-Bazi A. (2021). Management of renewable energy production and distribution planning using agent-based modelling. Renew. Energy.

[3] Martinez-Anido C.B., Botor B., Florita A.R., Draxl C., Lu S., Hamann H.F., Hodge B.-M. (2016). The value of day-ahead solar power forecasting improvement. Solar Energy.

[4] Valentine K., Temple W., Thomas R.J., Zhang K.M. (2016). Relationship between wind power, electric vehicles and charger infrastructure in a two-settlement energy market. Int. J. Electr. Power Energy Syst..

[5] Romero-Ruiz J., Pérez-Ruiz J., Martin S., Aguado J.A., la Torre S.D. (2016). Probabilistic congestion management using EVs in a smart grid with intermittent renewable generation. Electr. Power Syst. Res..

[6] Ahmad S., Ahmad A., Naeem M., Ejaz W., Kim H. (2018). A Compendium of Performance Metrics, Pricing Schemes, Optimization Objectives, and Solution Methodologies of Demand Side Management for the Smart Grid. Energies.

[7] Malik S., Harish V.S.K.V. (2019). Integration of automated Demand Response and Energy Efficiency to enable a smart grid infrastructure. Proceedings of the 2019 2nd International Conference on Power Energy, Environment and Intelligent Control (PEEIC).

[8] Ghatikar G., Zuber J., Koch E., Bienert R. (2014). Smart grid and customer transactions: The unrealized benefits of conformance. Proceedings of the 2014 IEEE Green Energy and Systems Conference (IGESC).

[9] Huh J.-H., Seo Y.-S. (2019). Understanding Edge Computing: Engineering Evolution with Artificial Intelligence. IEEE Access.

[10] Giraldo-Soto C., Erkoreka A., Mora L., Uriarte I., Portillo L.D. (2018). Monitoring System Analysis for Evaluating a Building’s Envelope Energy Performance through Estimation of Its Heat Loss Coefficient. Sensors.

[11] Parejo A., Sanchez-Squella A., Barraza R., Yanine F., Barrueto-Guzman A., Leon C. (2019). Design and Simulation of an Energy Homeostaticity System for Electric and Thermal Power Management in a Building with Smart Microgrid. Energies.

[12] Cheng Z., Li Z., Liang J., Si J., Dong L., Gao J. (2020). Distributed coordination control strategy for multiple residential solar PV systems in distribution networks. Int. J. Electr. Power Energy Syst..

[13] Papavasiliou A., Mezghani I. (2018). Coordination Schemes for the Integration of Transmission and Distribution System Operations. Proceedings of the 2018 Power Systems Computation Conference (PSCC).

[14] Migliavacca G., Rossi M., Gerard H., Dzamarija M., Horsmanheimo S., Madina C., Kockar I., Leclecq G., Marroquín M., Svendsen H.G. TSO-DSO coordination and market architectures for an integrated ancillary services acquisition: The view of the SmartNet project. Proceedings of the CIGRE Session 2018.

[15] European Union (2019). Directive (EU) 2019/ 944 of The European Parliament and of The Council—of 5 June 2019—on Common Rules for the Internal Market for Electricity and Amending Directive 2012/27/EU.75.

[16] Alonso J.I.G., Personal E., García S., Parejo A., Rossi M., García A., Delfino F., Pérez R., León C. (2020). Flexibility Services Based on OpenADR Protocol for DSO Level. Sensors.

[17] Yanine F., Sanchez-Squella A., Barrueto A., Sahoo S.K., Parejo A., Shah D., Cordova F.M. (2019). Homeostaticity of energy systems: How to engineer grid flexibility and why should electric utilities care. Period. Eng. Nat. Sci..

[18] Mak S.T., So E. (2014). Integration of PMU, SCADA, AMI to accomplish expanded functional capabilities of Smart Grid. Proceedings of the 29th Conference on Precision Electromagnetic Measurements (CPEM 2014).

[19] Huh J.-H. (2018). Smart Grid Test Bed Using OPNET and Power Line Communication.

[20] European Comission Benchmarking Smart Metering Deployment in the EU-28. Final Report. March 2020. https://op.europa.eu/en/publication-detail/-/publication/b397ef73-698f-11ea-b735-01aa75ed71a1.

[21] European Parliamen (2009). Directive 2009/72/EC of the European Parliament and of the Council of 13 July 2009 Concerning Common Rules for the Internal Market in Electricity and Repealing Directive 2003/54/EC 2009.

[22] Cui T., Carr J., Brissette A., Ragaini E. (2017). Connecting the Last Mile: Demand Response in Smart Buildings. Energy Procedia.

[23] Ren H., Ortega J., Casimis D.W. (2017). Review of Operating Reserves and Day-Ahead Unit Commitment Considering Variable Renewable Energies: International Experience. IEEE Lat. Am. Trans..

[24] San Diego Gas & Electric Company (2019). Schedule CBP: Capacity Bidding Program.

[25] Pacific Gas & Electric Company (2018). Electric Schedule E-CBP: Capacity Bidding Program.

[26] Guerrero J.I., Personal E., Caro S.G., Parejo A., Rossi M., Garcia A., Sanchez R.P., Leon C. (2020). Evaluating Distribution System Operators: Automated Demand Response and Distributed Energy Resources in the Flexibility4Chile Project. IEEE Power Energy Mag..

[27] Samad T., Koch E. (2012). Automated demand response for energy efficiency and emissions reduction. PES T&D 2012.

[28] Samad T., Koch E., Stluka P. (2016). Automated Demand Response for Smart Buildings and Microgrids: The State of the Practice and Research Challenges. Proc. IEEE.

[29] OpenADR Alliance (2013). OpenADR 2.0 Profile Specification B Profile. https://www.openadr.org/specification.

[30] Kiliccote S., Dudley J.H., Piette M.A. (2009). Northwest Open Automated Demand Response Technology Demonstration Project.

[31] McParland C. (2011). OpenADR open source toolkit: Developing open source software for the Smart Grid. Proceedings of the 2011 IEEE Power and Energy Society General Meeting.

[32] Herberg U., Mashima D., Jetcheva J.G., Mirzazad-Barijough S. (2014). OpenADR 2.0 deployment architectures: Options and implications. Proceedings of the 2014 IEEE International Conference on Smart Grid Communications (SmartGridComm).

[33] Jingxi Z., Min C., Ling W., Lixia Y., Shixiong P., Dong L. (2018). Research on Architecture of Automatic Demand Response System Based on OpenADR. Proceedings of the 2018 China International Conference on Electricity Distribution (CICED).

[34] Wilcox J., Kaleshi D., Sooriyabandara M. (2014). DIRECTOR: A distributed communication transport manager for the Smart Grid. Proceedings of the 2014 IEEE International Conference on Communications (ICC).

[35] Koch E.L. (2012). Automated demand response—mathsemicolonfrom peak shaving to ancillary services. Proceedings of the 2012 IEEE PES Innovative Smart Grid Technologies (ISGT).

[36] Goli S., McKane A., Olsen D. (2012). Demand Response Opportunities in Industrial Refrigerated Warehouses in California.

[37] Seo J., Jin J., Kim J.Y., Lee J.-J. (2016). Automated Residential Demand Response Based on Advanced Metering Infrastructure Network. Int. J. Distrib. Sens. Netw..

[38] Parejo A., Garcia S., Personal E., Garcia A., Guerrero J.I., Leon C. (2020). Living-Lab for Smart Grid technologies teaching. Proceedings of the 2020 XIV Technologies Applied to Electronics Teaching Conference (TAEE).

[39] Fagiani M., Squartini S., Gabrielli L., Spinsante S., Piazza F. (2015). A review of datasets and load forecasting techniques for smart natural gas and water grids: Analysis and experiments. Neurocomputing.

[40] Daut M.A.M., Hassan M.Y., Abdullah H., Rahman H.A., Abdullah M.P., Hussin F. (2017). Building electrical energy consumption forecasting analysis using conventional and artificial intelligence methods: A review. Renew. Sustain. Energy Rev..

[41] Deb C., Zhang F., Yang J., Lee S.E., Shah K.W. (2017). A review on time series forecasting techniques for building energy consumption. Renew. Sustain. Energy Rev..

[42] Piette M.A., Kiliccote S., Ghatikar G., McKane A.T., Matson N., Page J., MacDonald J.S., Aghajanzadeh A., Black D.R., Yin R. (2015). Demand Response Research Center—Final Report.

[43] Electric Power and Research Institute (EPRI) (2017). OpenADR 2.0b Open Source Virtual Top Node (OADR2.0b VTN) Version 0.9.7.0..

[44] Du Y.F., Jiang L., Duan C., Li Y.Z., Smith J.S. (2018). Energy Consumption Scheduling of HVAC Considering Weather Forecast Error Through the Distributionally Robust Approach. IEEE Trans. Ind. Inform..

[45] Bianco V., Piazza G., Scarpa F., Tagliafico L.A. (2017). Energy, economic and environmental assessment of the utilization of heat pumps for buildings heating in the Italian residential sector. Int. J. Heat Technol..

[46] Hosseinzadeh M., Salmasi F.R. (2015). Robust Optimal Power Management System for a Hybrid AC/DC Micro-Grid. IEEE Trans. Sustain. Energy.

[47] Lagrange A., de Simón-Martín M., González-Martínez A., Bracco S., Rosales-Asensio E. (2020). Sustainable microgrids with energy storage as a means to increase power resilience in critical facilities: An application to a hospital. Int. J. Electr. Power Energy Syst..

[48] Huh J.-H., Otgonchimeg S., Seo K. (2016). Advanced metering infrastructure design and test bed experiment using intelligent agents: Focusing on the PLC network base technology for Smart Grid system. J. Supercomput..

[49] IPKeys Technologies (2017). EISS Box 3.0 System/Device Monitoring and Management.

